# Association of Residential Proximity to the Coast With Incident Myocardial Infarction: A Prospective Cohort Study

**DOI:** 10.3389/fcvm.2022.752964

**Published:** 2022-02-17

**Authors:** Zhuang Xiao-dong, Zhang Shao-zhao, Hu Xun, Liao Xin-xue, Liao Li-zhen

**Affiliations:** ^1^Department of Cardiology, The First Affiliated Hospital of Sun Yat-sen University, Guangzhou, China; ^2^NHC Key Laboratory of Assisted Circulation, Sun Yat-sen University, Guangzhou, China; ^3^Health Department, Guangdong Provincial Key Laboratory of Pharmaceutical Bioactive Substances, Guangzhou Higher Education Mega Center, Guangdong Pharmaceutical University, Guangzhou, China; ^4^Guangdong Engineering Research Center for Light and Health, Guangzhou Higher Education Mega Center, Guangzhou, China

**Keywords:** myocardial infarction, distance to coast, cohort, UK Biobank, association

## Abstract

**Background:**

Little is known about how the residential distance to the coast is associated with incident myocardial infarction (MI) and which mechanisms may explain the association. We aim to explore this association using data from a prospective, population-based cohort with unprecedented sample size, and broad geographical coverage.

**Methods:**

In this study, 377,340 participants from the UK Biobank were included.

**Results:**

It was shown that 4,059 MI occurred during a median 8.0 years follow-up. Using group (<1 km) as reference, group (20–50 km) was associated with a lower risk of MI (hazard ratio, *HR* 0.79, 95% *CI* 0.64–0.98) and a *U*-shaped relation between distance to the coast and MI was shown with the low-risk interval between 32 and 64 km (*p*_non−linear_ = 0.0012). Using participants of the intermediate region (32–64 km) as a reference, participants of the offshore region (<32 km) and inland region (>64 km) were both associated with a higher risk of incident MI (*HR* 1.12, 95% *CI* 1.04–1.21 and *HR* 1.09, 95% *CI* 1.01–1.18, respectively). *HR* for offshore region (<32 km) was larger in subgroup with low total physical activity (<24 h/week) (*HR* 1.24, 95% *CI* 1.09–1.42, *p*_interaction_ = 0.043). *HR* for inland region (>64 km) was larger in subgroup in urban area (*HR* 1.12, 95% *CI* 1.03–1.22, *p*_interaction_ = 0.065) and in subgroup of high nitrogen dioxide (NO_2_) air pollution (*HR* 1.29, 95% *CI* 1.11–1.50, *p*_interaction_ = 0.021).

**Conclusion:**

We found a *U*-shaped association between residential distance to the coast and incident MI, and the association was modified by physical activity, population density, and air pollution.

## Keypoints

Question

- Proximity to the coast, an essential natural outdoor environment attribute, is positively related to self-reported general and mental health.

Findings

- We found a *U*-shaped association between residential distance to the coast and incident myocardial infarction (MI). The association of offshore region with incident MI was modified by total physical activity. The association of inland region with incident MI was modified by urban/rural area or nitrogen dioxide (NO_2_) air pollution.

Meaning

- The harmful effect of residential distance to coast on incident MI may vary with the distance of coastline and is regulated by various factors. Therefore, when possible, advice on the living environment and health should be personalized.

## Background

Natural outdoor environment attributes, such as green spaces (i.e., forests or parks), blue spaces (i.e., visible bodies of water), and coastal proximity, have long-term effects on behavior and health (1–3). Researchers have provided preliminary evidence that proximity to the coast, an essential natural outdoor environment attribute, is positively related to both self-reported general and mental health, and the beneficial effect of coastal proximity was mainly mediated by improving behavioral pathways (such as physical activity, sleep, and diet), alleviating stress, and avoiding environmental pollution (4–6). However, most of these studies are limited by a small sample size, weak geographical representation, insufficient adjustment for confounders, and unclear definition of exposure. Besides, most of the outcomes of previous reports were self-reported and not relating to specific diseases. Hence, little is known about how the residential distance to the coast is associated with the incidence of myocardial infarction (MI) and which mechanisms may explain the association.

To deal with these limitations, we explored the association between distance to the coast and incident MI using data from UK Biobank, a prospective, population-based cohort study with unprecedented sample size and broad geographical coverage.

## Methods

### Study Population

UK Biobank is a large prospective cohort of middle-aged adults designed to support biomedical analysis focused on improving the prevention, diagnosis, and treatment of chronic disease, the methods and aim of which have been reported elsewhere (7). In brief, between April 2007 and December 2010, UK Biobank recruited 502,628 participants (5.5% response rate, most of whom were age 40–70 years) from the general population (8). Participants attended 1 of 22 assessment centers across England, Wales, and Scotland and completed a touch screen questionnaire, had physical measurements taken, and provided biological samples. All participants provided written informed consent, and the study was approved by the NHS National Research Ethics Service. This research has been conducted using the UK Biobank Resource under Application Number 56,925.

In the present study, we included participants with data of distance from participant's residence location to the coast (*n* = 440,874), excluded participants with previous cardiovascular diseases (CVDs) (coronary heart disease and stroke, *n* = 28,980) or cancer (*n* = 34,544) at baseline, leaving 377,340 participants remained for analysis (Supplementary Figure S1).

### Ascertainment of Outcome

In UK Biobank, hospital admissions were identified *via* record linkage to Health Episode Statistics records for England and Wales and the Scottish Mortality Records for Scotland (8). Detailed information about recorded linkage procedures is available online. Incident MI, comprising fatal and non-fatal ST-segment elevation and non-ST-segment elevation MI, was defined as ICD 10 (international classification of diseases, 10th revision) code of I21, I21.4, and I21.9 recorded on hospital admission. At the time of analysis, the last recorded MI was on March 31, 2017, which was used as the censoring date for other participants if no outcome had been recorded, whichever occurred first.

### Ascertainment of Exposures

Environmental indicators attributed to participants were based on home location grid references. Data on the natural environment were linked using CEH 2007 Land Cover Map data. Measures of residential greenspace were estimated for England residents using the 2005 Generalized Land Use Database for England. It provides data on land use distribution for 2001 Census Output Areas in England and is consistent with the previous related research (9, 10). Residential distance to the coast was defined as the participant's residence location to the coast according to the participant's address, measured in Kilometers (km). The Euclidean distance raster from the coastline was calculated for a small grid cell size, then values from the grid allocated to UKB point locations. Based on existing literature, distances to the coast were collapsed into five categories: 0–1 km, 1–5 km, 5–20 km, 20–50 km, and over 50 km (11). To obtain approximately equal sample sizes per category, we divided the data into five quintiles for the current analyzes.

### Data on Potential Confounders and Effect Modifiers

Sociodemographic factors (age, gender, ethnicity, Townsend deprivation index, professional qualifications, income, employment, and month of recruitment), health-related variables (overall health rating, mental health, handgrip strength, family history of heart diseases, medication for aspirin, cholesterol, and blood pressure, prevalent diabetes, and hypertension at baseline), lifestyle factors (smoking status, drinking status, body mass index, total physical activity, sedentary lifestyle, sleep duration, and dietary intake), residential air and noise pollution (nitrogen oxides, nitrogen dioxide [NO_2_], particulate matter [PM], traffic intensity, average daytime/night sound level of noise pollution), home area population density classified as urban or rural, and greenspace (domestic garden percentage, greenspace percentage, natural environment percentage, and water percentage) were treated as potential confounders.

Age was calculated from dates of birth and baseline assessment. Qualification, average total household income, current employment status, overall health rating, mental health status, family history of heart diseases, the medication used, and sleep pattern were recorded using an electronic questionnaire completed by participants. Smoking status and drinking status were categorized into never, former and current smoker or drinker. Area-based socioeconomic status was derived from the postal code of residence by using the Townsend deprivation score (12). Dietary information was collected *via* the Oxford WebQ; a web-based 24 recall questionnaire developed specifically for large population studies (13). Physical activity was based on self-report by using the International Physical Activity Questionnaire short form, and total physical activity was calculated as the sum of walking, moderate, and vigorous exercise measured as metabolic equivalents (MET-h/week) (14). Grip strength was accessed through the use of a hydraulic hand dynamometer while sitting (14, 15). Total time spent in sedentary behaviors was derived from the sum of self-reported time spent driving, using a computer, and watching television. Land use regression (LUR)-based estimates of NO_2_, PM10, and PM2.5 for 2010 were generated as part of the European Study of Cohorts for Air Pollution Effects (ESCAPE) and link to geocoded residential addresses of UK Biobank participants (16). Noise estimates were derived from a simplified version of the Common Noise Assessment Methods in the European Union (CNOSSOS-EU) framework (17). Home area population density classified as urban or rural was derived by combining each participant's home postcode with data generated from the 2001 census from the Office of National Statistics, using the Geoconvert tool from Census Dissemination Unit. More details for each variable are available on the UK Biobank website http://www.ukbiobank.ac.uk/.

### Statistical Analysis

Baseline characteristics of 377,340 participants were described as means or percentages and were compared between groups using the one-way ANOVA test, the χ2 test, and the Kruskal–Wallis test, as appropriate. We coded missing data as a missing indicator category for categorical variables, such as smoking status, and mean values for continuous variables.

The association between residential distance to coast and MI was explored using Cox proportional hazard models. The proportional hazard assumption was checked by tests based on Schoenfeld residuals. The results were reported as hazard ratios (*HRs*) together with 95% *CIs*. First, distance to the coast was treated as continuous variables, and *HRs* were calculated per 1 SD (26.7 km) difference in distance to the coast. Then we categorized the distance to coast into <1 km, 1–5 km, 5–20 km, 20–50 km, and ≥50 km groups and calculated the *HRs* for the other four groups taking the first group (<1 km) as reference. We also categorized distance to coast into quintiles (Q1–Q5) base on the sample distribution and calculated the HRs for the last four groups taking the first quintile (Q1) as reference. Models were arranged a priori to investigate the impact of incremental adjustment. Model 1 adjusted for age, gender, ethnicity, social deprivation, income, employment status, total physical activity, overall health rating, smoking, drinking status, BMI, and handgrip strength. Model 2 additionally adjusted for family history of heart diseases, medication for aspirin, cholesterol, and blood pressure, prevalent diabetes, and hypertension. Model 3 further adjusted for air pollution, noise pollution, sleep duration, dietary intake, and home area population density.

To examine the overall statistical significance and the non-linearity of the exposure, we used likelihood ratio tests. A multivariable restricted cubic spline with 3 knots was used to express the dose-response relationship. We calculated *HRs* for living in the offshore region (<32 km) and inland region (>64 km) using Cox-proportional hazard models with incremental adjustment separately, taking participants in the intermediate area (32–64 km) within the lowest risk interval as a reference, according to the result of the restricted cubic spline. We conducted subgroup analyses to assess potential modification effects by the following factors: sex, age, BMI, sedentary behavior, sleep duration, total physical activity, smoking status, drinking status, income, area-based socioeconomic status, mental health status, urban area, air pollution, noise pollution, and hypertension. A sensitivity analysis was also conducted to investigate the effect of removing MI occurring within the first 24 months of follow-up to reduce the possible impact of reverse causation. Effect modifiers were investigated by adding to the fully adjusted model an interaction term between exposure and each of these variables.

All analyses were performed with SPSS V26 (IBM) and Stata V15 (Stata Corporation, College Station, TX, USA). A two-sided value of *p* < 0.05 was considered statistically significant.

## Results

The baseline characteristics of the participants are summarized in Table 1. The mean value of residential distance to the coast for 377,340 participants was 45.7 ± 26.7 km. Briefly, participants who lived proximal to the coast (<1 km) were more likely to be white, retired, less social deprivation, less diabetes, more physically active, and exposed to less NO_2_ air pollution and traffic intensity. Supplementary Table S1 shows the characteristics of participants by quintiles (Q1–Q5) of residential distance to the coast.

**Table 1:** The baseline characteristics of the participants by residential distance to the coast.

**Characteristic**	**Total** **(*n* = 377,340)**	**Distance to coast, km**					***P*-value**
		**≤1 km** **(*n* = 6,893)**	**1–5 km** **(*n* = 19,402)**	**5–20 km** **(*n* = 68,104)**	**20–50 km** **(*n* = 96,586)**	**>50 km** **(*n* = 186,355)**	
Distance to coast, km	45.7 ± 26.7	0.6 ± 0.3	2.6 ± 1.1	11.6 ± 3.8	37.5 ± 8.0	68.6 ± 12.2	<0.001
Male	169,741 (45.0%)	3,072 (44.6%)	8,558 (44.1%)	30,205 (44.4%)	43,112 (44.6%)	84,794 (45.5%)	<0.001
Age, years	55.9 ± 8.1	56.7 ± 7.9	56.4 ± 8.1	55.6 ± 8.2	56.0 ± 8.1	55.9 ± 8.1	<0.001
White	352,453 (93.4%)	6,793 (98.5%)	19,083 (98.4%)	66,106 (97.1%)	87,157 (90.2%)	173,314 (93.0%)	<0.001
Education							<0.001
College or University degree	122,650 (32.5%)	2,110 (30.6%)	4,628 (23.9%)	19,548 (28.7%)	37,293 (38.6%)	59,071 (31.7%)	
A levels/AS levels	42,306 (11.2%)	799 (11.6%)	2,236 (11.5%)	7,382 (10.8%)	11,294 (11.7%)	20,595 (11.1%)	
O levels/GCSEs	80,726 (21.4%)	1,608 (23.3%)	4,834 (24.9%)	15,765 (23.1%)	18,775 (19.4%)	39,744 (21.3%)	
CSEs or other	65,306 (17.3%)	1,323 (19.2%)	4,041 (20.8%)	13,158 (19.3%)	14,943 (15.5%)	31,841 (17.1%)	
None	66,352 (17.6%)	1,053 (15.3%)	3,663 (18.9%)	12,251 (18.0%)	14,281 (14.8%)	35,104 (18.8%)	
Income, £							<0.001
<18,000	125,367 (33.2%)	2,152 (31.2%)	7,013 (36.1%)	22,610 (33.2%)	29,810 (30.9%)	63,782 (34.2%)	
18,000–30,999	80,207 (21.3%)	1,573 (22.8%)	4,553 (23.5%)	15,351 (22.5%)	19,149 (19.8%)	39,581 (21.2%)	
31,000–51,999	85,267 (22.6%)	1,620 (23.5%)	4,549 (23.4%)	15,929 (23.4%)	21,515 (22.3%)	41,654 (22.4%)	
52,000–100,000	68,140 (18.1%)	1,305 (18.9%)	2,857 (14.7%)	11,880 (17.4%)	18,996 (19.7%)	33,102 (17.8%)	
>100,000	18,359 (4.9%)	243 (3.5%)	430 (2.2%)	2,334 (3.4%)	7,116 (7.4%)	8,236 (4.4%)	
Current employment status							<0.001
In paid employment	227,225 (60.2%)	3880 (56.3%)	11,012 (56.8%)	411.83 (60.5%)	58,411 (60.5%)	112,739 (60.5%)	
Retired	115,064 (30.5%)	2,457 (35.6%)	6,594 (34.0%)	20,703 (30.4%)	28,380 (29.4%)	56,930 (30.5%)	
Looking after home	11,055 (2.9%)	195 (2.8%)	549 (2.8%)	1,995 (2.9%)	3,053 (3.2%)	5,263 (2.8%)	
Others	23,996 (6.4%)	361 (5.2%)	1,247 (6.4%)	4,223 (6.2%)	6,742 (7.0%)	11,423 (6.1%)	
Drinking status							<0.001
Never	17,831 (4.7%)	228 (3.3%)	694 (3.6%)	2,587 (3.8%)	4,814 (5.0%)	9,508 (5.1%)	
Previous	12,506 (3.3%)	220 (3.2%)	646 (3.3%)	2,269 (3.3%)	3,311 (3.4%)	6,060 (3.3%)	
Current	347,003 (92.0%)	6,445 (93.5%)	18,062 (93.1%)	63,248 (92.9%)	88,461 (91.6%)	170,787 (91.6%)	
Smoking status							<0.001
Never	212,622 (56.3%)	3,910 (56.7%)	10,999 (56.7%)	38,124 (56.0%)	53,350 (55.2%)	106,239 (57.0%)	
Previous	125,960 (33.4%)	2,367 (34.3%)	6,456 (33.3%)	22,972 (33.7%)	32.860 (34.0%)	61,305 (32.9%)	
Current	38,758 (10.3%)	616 (8.9%)	1,947 (10.0%)	7,008 (10.3%)	10,376 (10.7%)	18,811 (10.1%)	
BMI, kg/m^2^	27.3 ± 4.7	27.4 ± 4.7	27.6 ± 4.7	27.4 ± 4.8	27.1 ± 4.7	27.3 ± 4.7	<0.001
Waist-hip ratio	0.87 ± 0.09	0.87 ± 0.09	0.87 ± 0.09	0.87 ± 0.09	0.87 ± 0.09	0.87 ± 0.09	<0.001
Total physical activity, hours/week	44.6 ± 40.7	45.6 ± 40.9	47.1 ± 43.1	45.4 ± 41.8	43.5 ± 40.7	44.5 ± 40.7	<0.001
Sedentary behavior, hours/day	4.5 ± 2.6	4.6 ± 2.5	4.8 ± 2.5	4.6 ± 2.6	4.5 ± 2.6	4.5 ± 2.5	<0.001
Home area							<0.001
Urban	323,552 (85.7%)	6,122 (88.8%)	17,039 (87.8%)	58,997 (86.6%)	84,105 (87.1%)	157,289 (84.4%)	
Rural	53,788 (14.3%)	771 (11.2%)	2,363 (12.2%)	9,107 (13.4%)	12,481 (12.9%)	29,066 (15.6%)	
Handgrip strength, Kg	29.6 ± 11.3	29.1 ± 11.1	29.1 ± 11.3	29.3 ± 11.3	29.7 ± 11.1	29.8 ± 11.3	<0.001
Townsend deprivation index	−1.3 ± 3.0	−1.7 ± 2.6	−1.5 ± 2.9	−1.5 ± 3.0	−0.6 ± 3.4	−1.6 ± 2.8	<0.001
Mental health	124,765 (33.1%)	2,372 (34.4%)	6,526 (33.6%)	23,130 (34.0%)	31,085 (32.2%)	61,652 (33.1%)	<0.001
Overall health rating							<0.001
Excellent	67,413 (17.9%)	1,210 (17.6%)	3,251 (16.8%)	12,011 (17.6%)	17,716 (18.3%)	33,225 (17.8%)	
Good	224,017 (59.4%)	4,210 (61.1%)	11,448 (59.0%)	40,310 (59.0%)	57,145 (59.2%)	110,904 (59.5%)	
Fair	73,088 (19.4%)	1,273 (18.5%)	3,967 (20.4%)	13,485 (19.8%)	18,462 (19.1%)	35,901 (19.3%)	
Poor	12,822 (3.4%)	200 (2.9%)	736 (3.8%)	2,298 (3.4%)	3,263 (3.4%)	6,325 (3.4%)	
Family history of heart diseases	144,592 (38.3%)	2,775 (40.3%)	7,698 (39.7%)	26,665 (39.2%)	36,132 (37.4%)	71,322 (38.3%)	<0.001
Hypertension	89,704 (23.8%)	1,614 (23.4%)	4,861 (25.1%)	16,121 (23.7%)	22,747 (23.6%)	44,361 (23.8%)	<0.001
Diabetes	13.626 (3.6%)	217 (3.1%)	632 (3.3%)	2,079 (3.1%)	3,674 (3.8%)	7,024 (3.8%)	<0.001
Aspirin	36,598 (9.7%)	692 (10.0%)	1,908 (9.8%)	6,431 (9.4%)	9,426 (9.8%)	18,141 (9.7%)	0.132
Anti-hypertension medicine	20,667 (5.5%)	385 (5.6%)	1,236 (6,4%)	3,743 (5.5%)	5,225 (5.4%)	10,078 (5.4%)	<0.001
Lipid-lowering medicine	21,138 (5.6%)	386 (5.6%)	1,224 (6.3%)	3.662 (5.4%)	5,850 (6.1%)	10,016 (5.4%)	<0.001
Nitrogen dioxide air pollution, microg/m^3^	26.7 ± 7.6	24.4 ± 6.0	26.0 ± 7.0	27.4 ± 7.3	28.7 ± 8.9	25.6 ± 6.8	<0.001
Nitrogen oxides, air pollution, micro/m^3^	44.0 ± 15.5	43.6 ± 13.8	44.7 ± 15.5	45.5 ± 14.8	47.3 ± 18.2	41.7 ± 13.8	<0.001
PM10, microg/m^3^	16.2 ± 1.9	15.9 ± 1.9	16.1 ± 1.7	16.3 ± 1.8	16.5 ± 2.0	16.1 ± 1.8	<0.001
PM2.5, microg/m^3^	10.0 ± 1.0	10.2 ± 1.1	10.3 ± 1.2	10.2 ± 1.1	10.2 ± 1.1	9.8 ± 0.9	<0.001
Traffic intensity on the nearest road, vehicles/day	1513.1 ± 4933.5	934.8 ± 2339.8	1228.2 ± 3815.1	1271.9 ± 4277.8	1764.7 ± 5606.4	1521.8 ± 4950.9	<0.001
Inverse distance to the nearest road, 1/meters	0.05 ± 0.07	0.05 ± 0.07	0.05 ± 0.07	0.05 ± 0.07	0.05 ± 0.07	0.05 ± 0.08	<0.001
Average daytime sound level of noise pollution, dB	55.4 ± 4.3	55.3 ± 3.7	55.3 ± 4.0	55.2 ± 4.0	55.9 ± 4.6	55.2 ± 4.2	<0.001
Average evening sound level of noise pollution, dB	51.7 ± 4.3	51.6 ± 3.7	51.5 ± 4.0	51.5 ± 4.0	52.1 ± 4.6	51.5 ± 4.2	<0.001
Average night-time sound level of noise pollution, dB	46.6 ± 4.3	46.5 ± 3.7	46.5 ± 4.0	46.4 ± 4.0	47.1 ± 4.6	46.4 ± 4.2	<0.001
Food weight, g	3263.1 ± 374.0	3268.4 ± 388.7	3258.9 ± 419.2	3264.1 ± 311.6	3262.8 ± 444.9	3263.1 ± 348.3	<0.001
Energy, KJ	8826.7 ± 1284.0	8836.9 ± 1277.4	8834.5 ± 1498.0	8835.6 ± 1065.4	8823.8 ± 1531.1	8823.8 ± 1189.4	<0.001
Protein, g	81.8 ± 12.4	81.9 ± 12.7	81.8 ± 13.9	81.9 ± 10.2	81.8 ± 14.9	81.8 ± 11.6	<0.001
Fat, g	76.8 ± 14.5	77.0 ± 15.2	76.9 ± 16.5	76.8 ± 12.1	76.8 ± 17.4	76.7 ± 13.4	<0.001
Carbohydrate, g	258.1 ± 43.1	258.1 ± 42.0	258.8 ± 50.0	258.4 ± 35.9	257.6 ± 51.4	258.3 ± 39.9	<0.001
Englyst dietary fiber, g	16.6 ± 3.1	16.7 ± 3.4	16.7 ± 3.5	16.7 ± 2.6	16.6 ± 3.7	16.6 ± 2.9	<0.001
Sleep duration, hours/day	7.14 ± 1.1	7.19 ± 1.1	7.15 ± 1.1	7.15 ± 1.1	7.13 ± 1.1	7.15 ± 1.1	<0.001

During a median of 8.0 years (3.0 million person-years) of follow-up, 4,059 cases of MI occurred. Table 2 shows the association between residential distance to the coast and incident MI. After adjusting potential confounders, no significant association with MI was observed for distance to coast as continuing variable (*HR* 0.98 per SD increase, 95% *CI* 0.95–1.02, *p* = 0.321). Using group (<1 km) as reference, group (20–50 km) was associated with a statistically significant lower risk of MI (*HR* 0.79, 95% *CI* 0.64–0.98, *p* = 0.033). When using the lowest quintile Q1 (<14.1 km) as reference, Q2 (14.1–40.2 km), and Q3 (40.2–56.4 km) were both associated with a statistically significant lower risk of MI (*HR* 0.86, 95% *CI* 0.78–0.95, *p* = 0.003; and *HR* 0.85, 95% *CI* 0.77–0.94, *p* = 0.001, respectively; *p* for trend = 0.586, Table 2).

**Table 2:** Association between distance to the coast and incident myocardial infarction (MI).

**Subgroup**	**Events rate**	**Model 1**		**Model 2**		**Model 3**		
		**HR (95% CI)**	***P*-value**	**HR (95% CI)**	***P*-value**	**HR (95% CI)**	***P*-value**	***P* for trend**
Per SD (26.7 km)	4,059/377,340	0.97 (0.94, 1.00)	0.085	0.97 (0.94, 1.00)	0.092	0.98 (0.95, 1.02)	0.321	
<1 km	94/6,893	Reference	-	Reference	-	Reference	-	
1–5 km	245/19,402	0.90 (0.71, 1.14)	0.372	0.89 (0.71, 1.13)	0.355	0.93 (0.73, 1.18)	0.564	
5–20 km	765/68,104	0.82 (0.66, 1.01)	0.067	0.82 (0.66, 1.01)	0.066	0.89 (0.71, 1.10)	0.280	
20–50 km	906/96,586	0.72 (0.58, 0.89)	0.002	0.72 (0.58, 0.89)	0.002	0.79 (0.64, 0.98)	0.033	
>50 km	2,049/186,355	0.77 (0.63, 0.95)	0.015	0.77 (0.63, 0.95)	0.015	0.84 (0.68, 1.04)	0.109	
Q1 (<14.1 km)	901/75,663	Reference	-	Reference	-	Reference	-	0.586
Q2 (14.1–40.2 km)	717/75,249	0.84 (0.76, 0.93)	0.001	0.84 (0.76, 0.93)	0.001	0.86 (0.78, 0.95)	0.003	
Q3 (40.2–56.4 km)	768/75,723	0.82 (0.75, 0.91)	<0.001	0.82 (0.75, 0.91)	<0.001	0.85 (0.77, 0.94)	0.001	
Q4 (56.4–70.2 km)	841/75,426	0.90 (0.82, 0.99)	0.033	0.90 (0.82, 0.99)	0.035	0.91 (0.83, 1.00)	0.058	
Q5 (≥70.2 km)	832/75,279	0.92 (0.84, 1.01)	0.079	0.92 (0.84, 1.01)	0.086	0.95 (0.86, 1.04)	0.265	

*Model 1: adjusted for age, gender, race, qualification, income, employ status, physical activity, sedentary behavior, mental health status, overall health rating, smoking status, drinking status, body mass index, waist to hip ratio, grip strength, and area-based socioeconomic status; Model 2: further adjusted for family history of heart diseases, the medication used of aspirin, antihypertension medicine and lip-lowing medicine, previous of hypertension and diabetes; Model 3: further adjusted for air pollution, noise pollution, dietary, sleep pattern, and home area population density*.

*HR, hazard ratio; CI, confident interval; SD, standard difference; Km, kilometer*.

**Figure 1:**
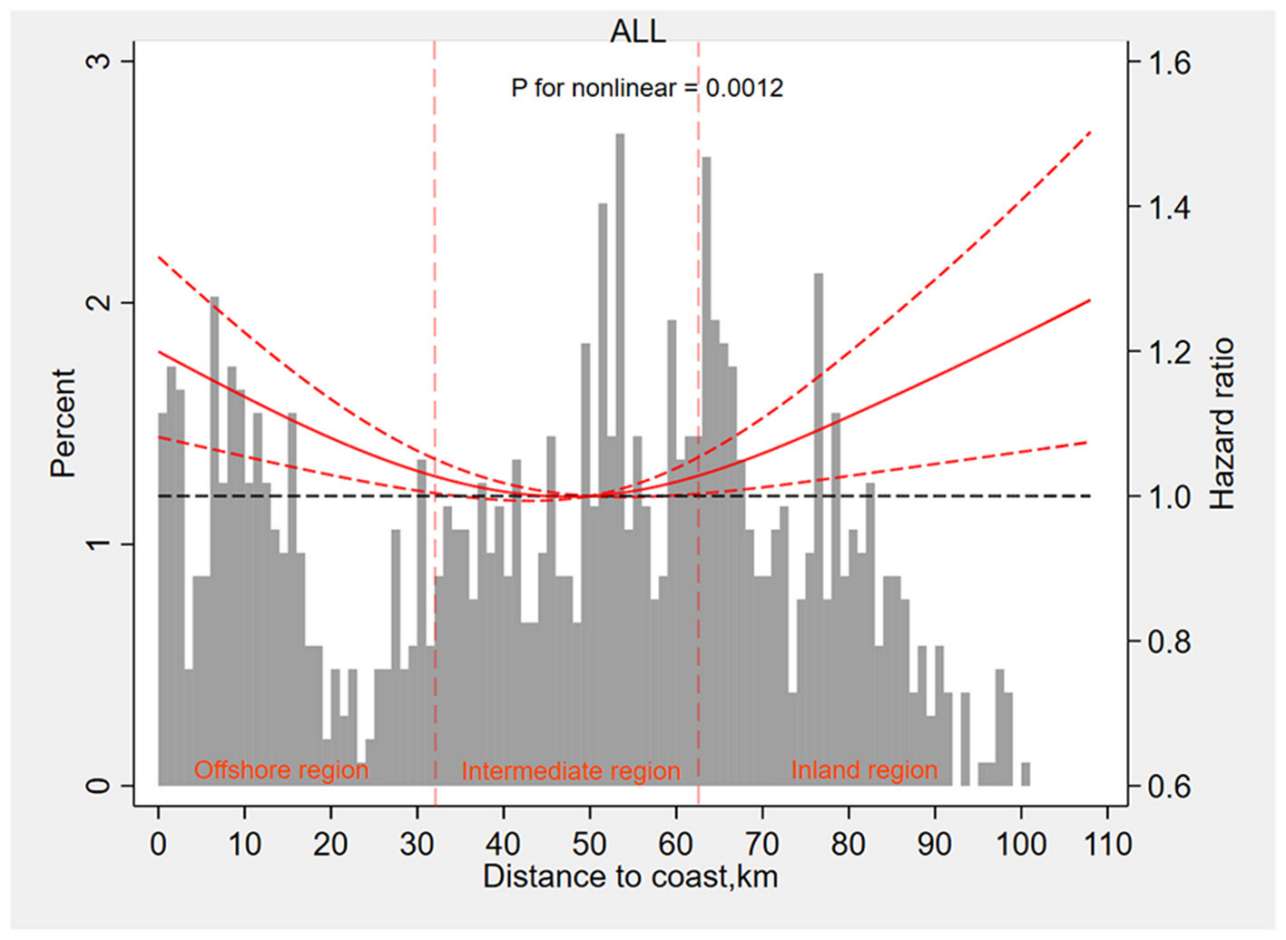
The restricted cubic spline curve to explore potential non-linear patterns between distance to the coast and myocardial infarction. Km, kilometers.

We then applied the restricted cubic spline curve to explore potential non-linear patterns. Figure 1 shows a *U*-shaped relation between distance to coast and MI with a relatively lower risk interval between 32 and 64 km (*p* for non-linear = 0.0012). According to the curve, we divided the population into three categories: offshore region (<32 km), inland region (>64 km), and intermediate area (32–64 km). Using participants of the intermediate region (32–64 km) as a reference, participants of the offshore region (<32 km), and inland region (>64 km) were both associated with a higher risk of incident MI and *HRs* were 1.12 (95% CI 1.04–1.21, *p* = 0.004) and 1.09 (95% *CI* 1.01–1.18, *p* = 0.027) after adjusting for all confounders, respectively (Table 3).

**Table 3:** Hazard ratio for participants of offshore region and inland region with MI.

**Space**	**Events rate**	**Model 1**		**Model 2**		**Model 3**	
		**HR (95% CI)**	***P*-value**	**HR (95% CI)**	***P*-value**	**HR (95% CI)**	***P*-value**
Offshore (<32 km)	2,866/268,449	1.16 (1.08, 1.25)	<0.001	1.16 (1.08, 1.25)	<0.001	1.12 (1.04, 1.21)	0.004
Inland (>64 km)	2,685/257,210	1.10 (1.02, 1.19)	0.014	1.10 (1.02, 1.19)	0.013	1.09 (1.01, 1.18)	0.027

*Model 1: adjusted for age, gender, race, qualification, income, employ status, physical activity, sedentary behavior, mental health status, overall health rating, smoking status, drinking status, BMI, waist to hip ratio, grip strength, and area-based socioeconomic status; Model 2: further adjusted for family history of heart diseases, the medication used of aspirin, antihypertension medicine and lip-lowing medicine, previous of hypertension and diabetes; Model 3: further adjusted for air pollution, noise pollution, dietary, sleep pattern, and home area population density*.

*HR, hazard ratio; CI, confident interval; Km, kilometer*.

**Figure 2:**
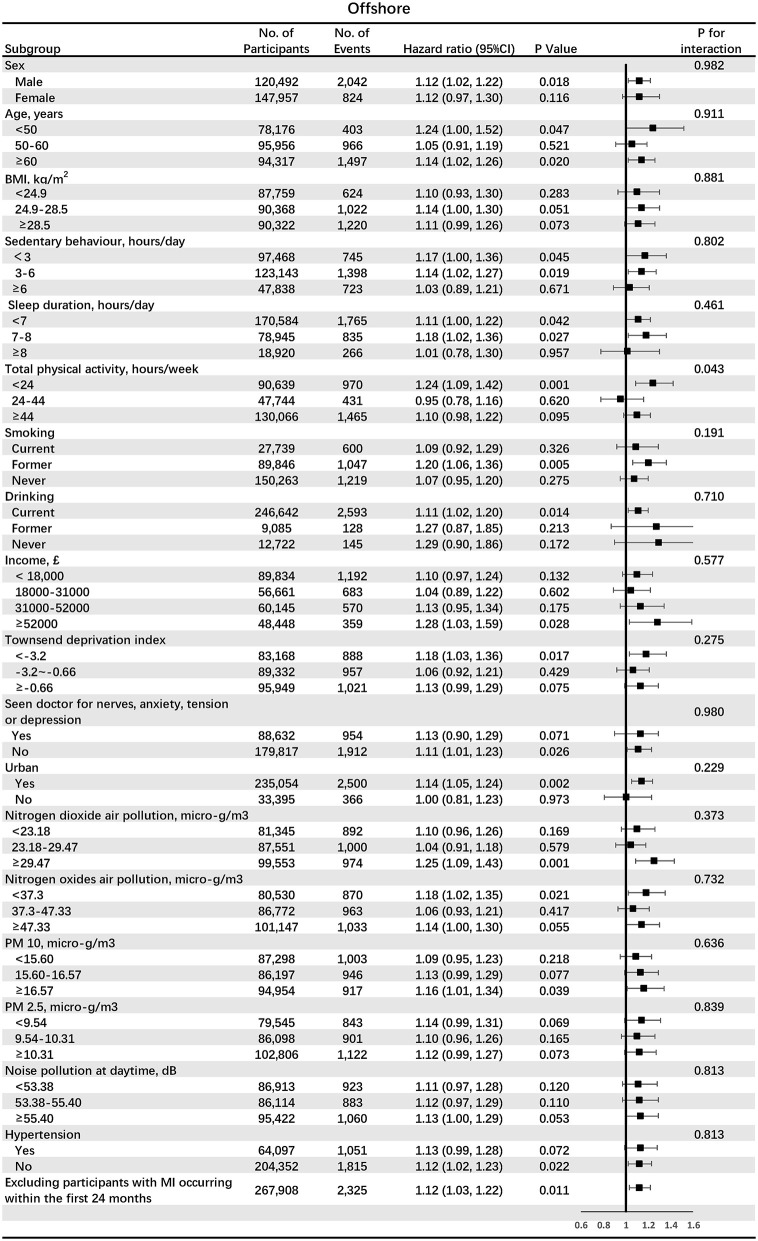
The associations between participants living in the offshore region (<32 km) and incident myocardial infarction (MI) in subgroups analyses. BMI, body mass index; PM, particulate matter.

**Figure 3:**
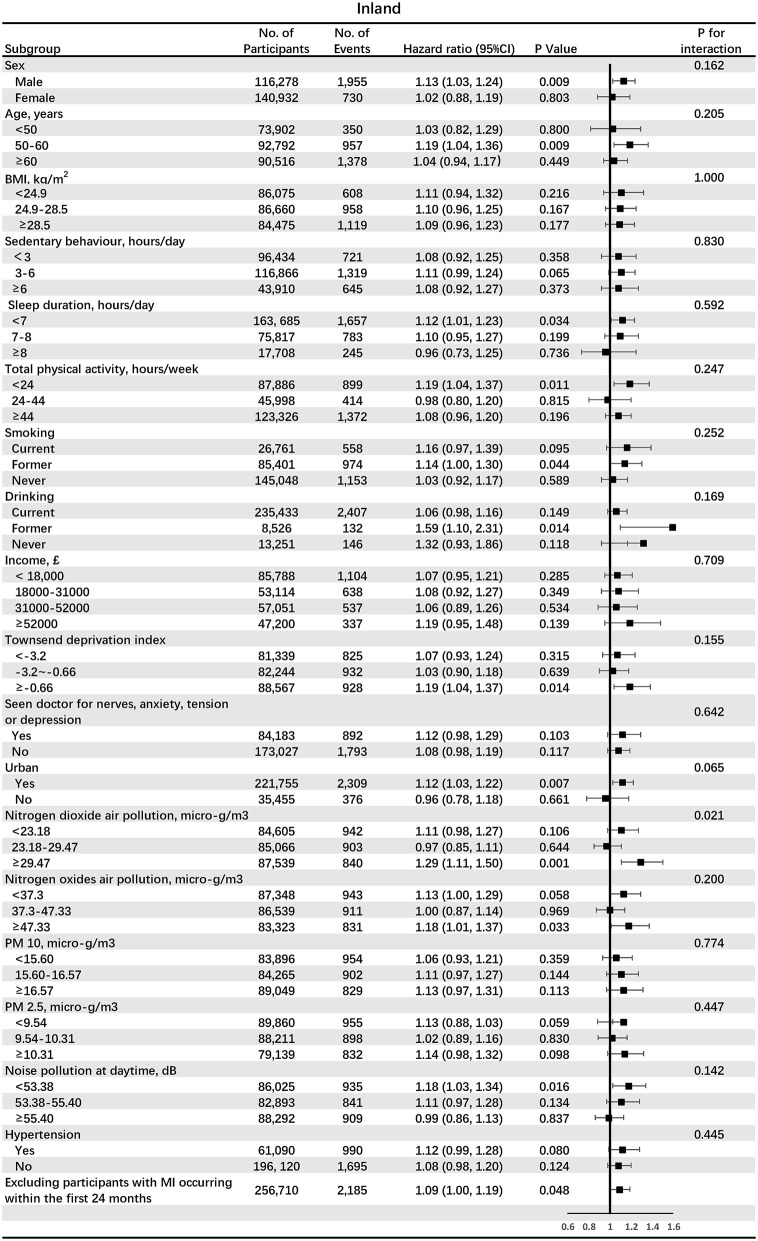
The associations between participants living in the inland region (>64 km) and incident MI in subgroups analyses. BMI, body mass index; PM, particulate matter.

Figure 2 shows the associations between participants living in the offshore region (<32 km) and incident MI in subgroups analyses. *HR* for the offshore area (<32 km) was higher in the subgroup with low total physical activity (<24 h/week) (*HR* 1.24, 95% *CI* 1.09–1.42, *p* = 0.001), compared with subgroups with moderate and high total physical activity (*p* for interaction = 0.043). Figure 3 shows the associations between participants living in the inland region (>64 km) and incident MI in subgroups stratified by potential effect modifiers. *HR* for the inland region (>64 km) was significantly larger in the subgroup of the urban area (*HR* 1.12, 95% *CI* 1.03–1.22, *p* = 0.007), compared with the subgroup of the rural area (*p* for interaction 0.065). Also, *HR* for the inland region (>64 km) was significantly larger in the subgroup of high NO_2_ air pollution exposure (*HR* 1.29, 95% *CI* 1.11–1.50, *p* = 0.001), compared with middle and low NO_2_ air pollution (*p* for interaction = 0.021).

## Discussion

**Figure 4:**
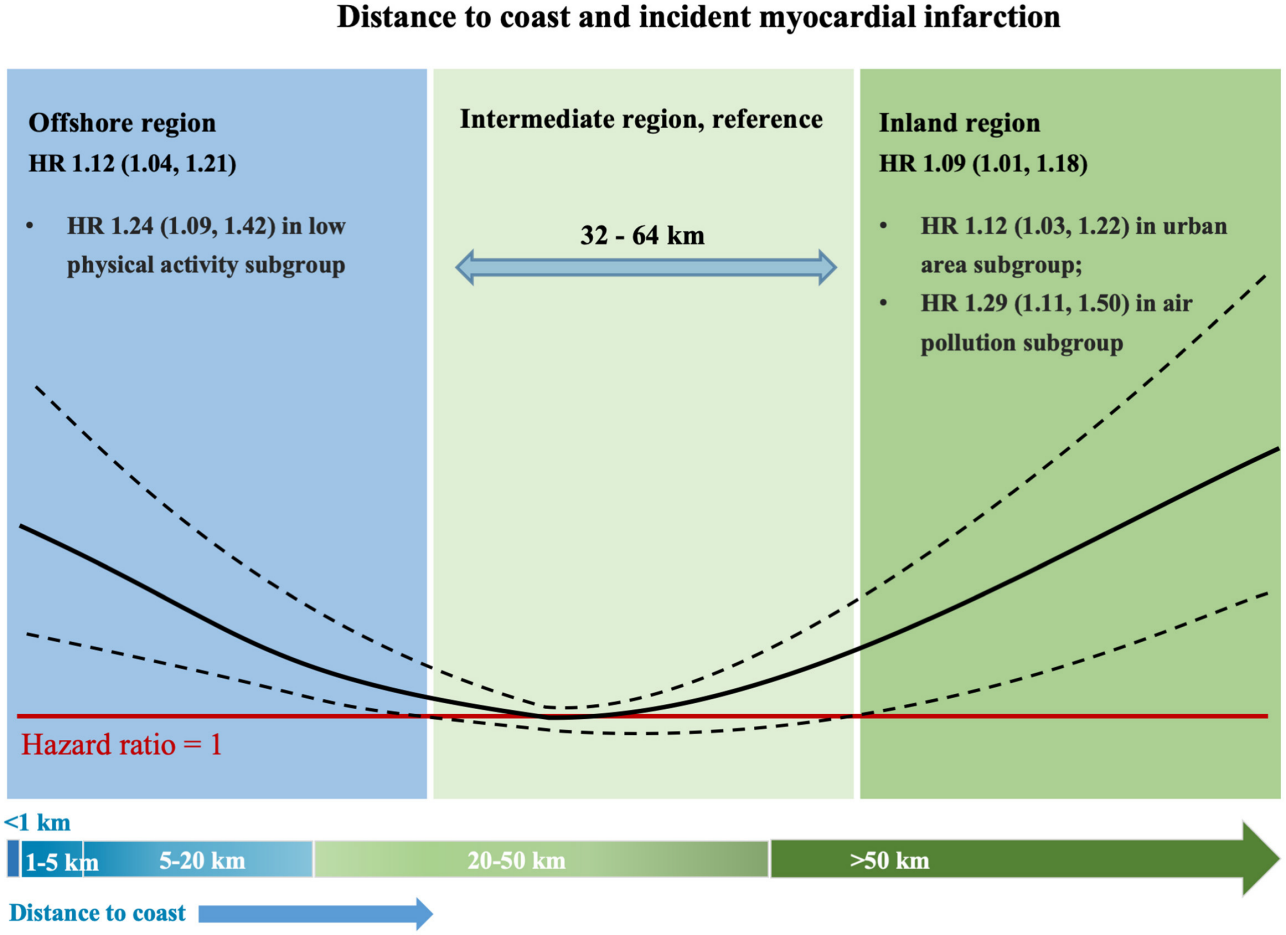
The non-linear pattern between distance to the coast and MI. HR, hazard ratio.

The main finding of the current study was that residential distance to coast had a *U*-shaped relation with incident MI in over 370,000 individuals followed for over 3.0 million person-years and that both residents of the offshore region (<32 km) and that of the inland region (>64 km) had around 10% increase risk of MI compared with residents of the intermediate region (32–64 km) (Figure 4). Moreover, the associations of offshore region and inland region with incident MI were modified by various factors. Participants of offshore regions with low total physical activity had a higher risk of MI (increased by 24%), suggesting that this subgroup may benefit from increased physical activity. Meanwhile, participants of inland regions living in the urban area or exposed to high NO_2_ air pollution had a higher risk of MI (increased by 12 and 29%, respectively), suggesting that urban environment improvement and air pollution control played a crucial role in these population. The results were mostly consistent in a series of sensitivity and subgroup analyses.

Unlike previous research that commonly reported linear associations, we investigated the non-linear association of coastal proximity with disease outcome. Our findings highlight the complex and diverse associations between residential distance to the coast and incident MI. Although modifying the residential environment tends to be problematic in the short term, our findings suggest that targeting based on coastal proximity and various effect modifiers (physical activity, population density, and air pollution) could help identify high-risk individuals and provide personalized interventions for diverse populations. However, the classification of the offshore, inland, and intermediate regions was constructed for illustrative purposes; hence the cut-off values of distance to coast identified in this study cannot be simply generalized. This classification is more about raising public awareness of the residential location than about being implemented as a real-world tool. The harmful effect of residential distance to coast on incident MI may vary with the coastline's distance and is regulated by various factors. Therefore, when possible, advice on the living environment and health should be personalized.

Scientific and public interest in the role of the natural outdoor environment in preventing CVDs is growing. A tremendous body of studies has provided evidence on the associations between various outdoor environment attributes, behavioral pathways, CV risk factors, and mortality (18–21). A meta-analysis of longitudinal studies found inverse relationships between neighborhood walkability and risk factors, such as obesity, hypertension, and type 2 diabetes mellitus (20). Similarly, another meta-analysis found that more residential greenspace was associated with reduced CVD mortality (22). The links between air and noise pollution, stress, and CVDs have also been recognized (23). The previous research suggested that living near and regularly visiting the coast or other large waterbodies was associated with better general and mental health. However, most of the outcomes used were self-reported and not disease-specific (24–26). However, the association of residential distance to the coast with CVD incidence is relatively less well studied, and high-quality epidemiological evidence was too scant to draw a conclusion. To investigate this issue at a broad level of disease specificity, we set out to address the question: does the incidence of MI increase or decrease with proximity to the coast?

The results of the study were somewhat unexpected, particularly the finding that MI risk increased in coastal areas, which contradicts most of the previous studies that have shown the benefits of living near the sea. However, our study was based on sufficient confounding adjustment and subgroup analyses, and the results were reliable and consistent. One possible explanation is that the same environment attribute might have diverse effects on cardiometabolic risk factors and different population subgroups. For example, whereas high population density might help improve the weight status through better availability of physical activity destinations and healthy food options, high population density might also have adverse effects on airway and CVD through more exposure to air pollution. Similarly, a previous study showed that individuals living in the <1 km coastal category had an average a 4 nmol/l higher vitamin D status compared to those living inland through increased solar irradiance, which can provide benefits in terms of vitamin D status but may also pose a risk due to higher skin cancer rates (27). Examining single environmental attributes with multiple behaviors and risk factors in single studies can provide insights into the differential effects of the natural environment on CVD. Further interdisciplinary research initiatives involving cardiology and urban design researchers must disentangle the complex relationships between the residential coastal distance and CVD.

Strengths of this study include its prospective design, a large sample size with harmonized exposure, health, and covariate data. We could adjust for a wide range of health, demographic, behavioral, and environmental confounders. The possibility of confounding was dealt with through statistical adjustment for a wide range of covariates, such as health, demographic, behavioral, and ecological confounders, and a series of sensitivity analyses. This study has several limitations. First, we were not able to consider the impact on the exposure of residential changes during follow-up, which will contribute to misclassification of long-term exposure relevant to the development of MI. These misclassifications are believed to be non-differential for cases, and non-cases likely bias the risk estimates toward the null. Second, as is the case for any observational study, residual confounding is always possible, and associations may not imply causation. We cannot rule out the possibility of residual confounding by other unaccounted factors, such as coastal climate, humidity, and sun exposure. Third, the UK Biobank represents the general population for age, sex, ethnicity, and deprivation within the age range recruited but is not representative in other regards, which may indicate a healthy volunteer selection bias. While this limits the ability to generalize prevalence rates, it should be possible to generalize the estimates of the associations' magnitude. Forth, the offshore, inland, and intermediate regions were constructed for illustrative purposes rather than as a tool ready for implementation. The cut-off values of distance to coast (32 km, 64 km) have not been validated.

## Conclusions

The study found a *U*-shaped association between residential distance to the coast and incident MI. Moreover, the association of offshore region with incident MI was modified by total physical activity. The association of inland region with incident MI was limited by urban/rural area or NO_2_ air pollution. Our findings highlight the complex and diverse associations between residential distance to the coast and incident MI, and residential advice should be personalized.

## Data Availability Statement

The datasets presented in this study can be found in online repositories. The names of the repository/repositories and accession number(s) can be found in the article/Supplementary Material.

## Author Contributions

ZX-d performed statistical analysis. LL-z handled funding and supervision. LX-x acquired the data. ZS-z conceived and designed the research. HX drafted the manuscript and made critical revision of the manuscript for critical intellectual content. All authors contributed to the article and approved the submitted version.

## Funding

The UK Biobank was supported by the Welcome Trust, Medical Research Council, Department of Health, Scottish Government, and the Northwest Regional Development Agency. It has also had funding from the Welsh Assembly Government and the British Heart Foundation. The research was designed, conducted, analyzed, and interpreted by the authors entirely independently of the funding sources.

## Conflict of Interest

The authors declare that the research was conducted in the absence of any commercial or financial relationships that could be construed as a potential conflict of interest.

## Publisher's Note

All claims expressed in this article are solely those of the authors and do not necessarily represent those of their affiliated organizations, or those of the publisher, the editors and the reviewers. Any product that may be evaluated in this article, or claim that may be made by its manufacturer, is not guaranteed or endorsed by the publisher.

## Abbreviations

MI: myocardial infarction
NO_2_: nitrogen dioxide
PM: particulate matter
HR: hazard ratios
Q1: first quintile
CVDs: cardiovascular diseases.
