# Supplementary material for: Association of Residential Proximity to the Coast With Incident Myocardial Infarction: A Prospective Cohort Study
Source: Front Cardiovasc Med. 2022 Feb 17;9:752964. doi: 10.3389/fcvm.2022.752964 (PMC8891518; doi:10.3389/fcvm.2022.752964)
Supplement: Supplementary file 1 [file Table_1.docx]

Table S1 The baseline characteristics of the participants by quintiles of residential distance to coast

| Characteristic | Total  (n=377,340) | Distance to coast, km | | | | | P value |
| --- | --- | --- | --- | --- | --- | --- | --- |
|  |  | Q1  (<14.1 km)  (n=75,663) | Q2  (14.1-40.2km)  (n=75,249) | Q3  (40.2-56.4 km)  (n=75,723) | Q4  (56.4-70.2 km)  (n=75,426) | Q5  (≥70.2 km)  (n=75,279) |  |
| Distance to coast, km | 45.7±26.7 | 7.1±4.2 | 28.1±8.2 | 49.1±4.6 | 63.3±3.7 | 81.1±7.5 | <0.001 |
| Male | 169,741 (45.0%) | 33,442 (44.2%) | 33,703 (44.8%) | 34,120 (45.1%) | 34,441 (45.7%) | 34,035 (45.2%) | <0.001 |
| Age, years | 55.9±8.1 | 55.8±8.2 | 56.0±8.1 | 55.8±8.1 | 55.9±8.1 | 56.2±8.1 | <0.001 |
| White | 352,453 (93.4%) | 73,625 (97.3%) | 68,257 (90.7%) | 68,511 (90.5%) | 72,034 (95.5%) | 70,026 (93.0%) | <0.001 |
| Education |  |  |  |  |  |  | <0.001 |
| College or University | 122,650 (32.5%) | 21,591 (28.5%) | 26,413 (35.1%) | 26,034 (34.4%) | 24,016 (31.8%) | 24,596 (32.7%) |  |
| A levels/AS levels | 42,306 (11.2%) | 8,386 (11,1%) | 8,548 (11.4%) | 8,542 (11.3%) | 8,585 (11.4%) | 8,245 (11.0%) |  |
| O levels/GCSEs | 80,726 (21.4%) | 17,487 (23.1%) | 15,671 (20.8%) | 15,082 (19.9%) | 16,590 (22.0%) | 15,896 (21.1%) |  |
| CSEs or other | 65,306 (17.3%) | 14,774 (19.5%) | 12,741 (16.9%) | 11,644 (15.4%) | 12,742 (16.9%) | 13.405 (17.9%) |  |
| None | 66,352 (17.6%) | 13,425 (17.7%) | 11,876 (15.8%) | 14,421 (19.0%) | 13,493 (17.9%) | 13,137 (17.5%) |  |
| Income, £ |  |  |  |  |  |  | <0.001 |
| < 18,000 | 125,367 (33.2%) | 25,656 (33.9%) | 23,801 (31.6%) | 26,100 (34.5%) | 24,355 (32.3%) | 25,455 (33.8%) |  |
| 18,000-30,999 | 80,207 (21.3%) | 17,058 (22.5%) | 15,962 (21.2%) | 14,584 (19.3%) | 16,343 (21.7%) | 16,260 (21.6%) |  |
| 31,000-51,999 | 85,267 (22.6%) | 17,596 (23.3%) | 17,124 (22.8%) | 16,109 (21.3%) | 17,282 (22.9%) | 17,156 (22.8%) |  |
| 52,000-100,000 | 68,140 (18.1%) | 12,877 (17.0%) | 13,932 (18.5%) | 14,198 (18.7%) | 13,969 (18.5%) | 13,164 (17.5%) |  |
| >100,000 | 18,359 (4.9%) | 2,476 (3.3%) | 4,430 (5.9%) | 4,732 (6.2%) | 3,477 (4.6%) | 3,244 (4.3%) |  |
| Current employment status |  |  |  |  |  |  | <0.001 |
| In paid employment | 227,225 (60.2%) | 45,095 (59.6%) | 45,080 (59.9%) | 45,980 (60.7%) | 46,241 (61.3%) | 44,829 (59.6%) |  |
| Retired | 115,064 (30.5%) | 23,482 (31.0%) | 22,670 (30.1%) | 22,209 (29.3%) | 23,093 (30.6%) | 23,610 (31.4%) |  |
| Looking after home | 11,055 (2.9%) | 2,208 (2.9%) | 2,214 (2.9%) | 2,485 (3.3%) | 2,022 (2.7%) | 2,126 (2.8%) |  |
| Others | 23,996 (6.4%) | 4,878 (6.4%) | 5,285 (7.0%) | 5,049 (6.7%) | 4,070 (5.4%) | 4,714 (6.3%) |  |
| Drinking status |  |  |  |  |  |  | <0.001 |
| Never | 17,831 (4.7%) | 2,776 (3.7%) | 3,821 (5.1%) | 4,197 (5.5%) | 3,101 (4.1%) | 3,936 (5.2%) |  |
| Previous | 12,506 (3.3%) | 2,550 (3.4%) | 2,652 (3.5%) | 2,550 (3.4%) | 2,293 (3.0%) | 2,461 (3.3%) |  |
| Current | 347,003 (92.0%) | 70,337 (93.0%) | 68,776 (91.4%) | 68,976 (91.1%) | 70,032 (92.8%) | 68,882 (91.5%) |  |
| Smoking status |  |  |  |  |  |  | <0.001 |
| Never | 212,622 (56.3%) | 42,166 (55,7%) | 41,889 (55,7%) | 42,516 (56.1%) | 43,001 (57.0%) | 43,040 (57.2%) |  |
| Previous | 125,960 (33.4%) | 25,602 (33.8%) | 25,415 (33.8%) | 25,092 (33.1%) | 25,037 (33.2%) | 24,814 (33.0%) |  |
| Current | 38,758 (10.3%) | 7,895 (10.4%) | 7,935 (10.5%) | 8,115 (10.7%) | 7,388 (9.8%) | 7,425 (9.9%) |  |
| BMI, kg/m^2^ | 27.3±4.7 | 27.4±4.8 | 27.3±4.7 | 27.2±4.7 | 27.2±4.7 | 27.3±4.7 | <0.001 |
| Waist-hip ratio | 0.87±0.09 | 0.87±0.09 | 0.87±0.09 | 0.87±0.09 | 0.87±0.09 | 0.87±0.09 | <0.001 |
| Total physical activity, hours/week | 44.6±40.7 | 45.8±41.9 | 44.2±40.3 | 43.6±39.2 | 44.9±41.3 | 44.4±40.8 | <0.001 |
| Sedentary behavior, hours/day | 4.5±2.6 | 4.6±2.6 | 4.5±2.6 | 4.5±2.6 | 4.5±2.5 | 4.5±2.5 | <0.001 |
| Home area |  |  |  |  |  |  | <0.001 |
| Urban | 323,552 (85.7%) | 67,276 (88.9%) | 62,930 (83.6%) | 68,797 (90.9%) | 62,648 (83.1%) | 61,901 (82.2%) |  |
| Rural | 53,788 (14.3%) | 8,387 (11.1%) | 12,319 (16.4%) | 6,926 (9.1%) | 12,778 (16.9%) | 13,378 (17.8%) |  |
| Hand grip strength, Kg | 29.6±11.3 | 29.2±11.3 | 29.5±11.1 | 29.6±11.3 | 30.2±11.4 | 29.5±11.2 | <0.001 |
| Townsend deprivation index | -1.3±3.0 | -1.3±3.0 | -0.7±3.5 | -1.1±3.0 | -1.9±2.7 | -1.6±2.8 | <0.001 |
| Seen doctor (GP) for nerves, anxiety, tension or depression | 124,765 (33.1%) | 25,753 (34.0%) | 24,588 (32.7%) | 24,330 (32.1%) | 25,122 (33.3%) | 24,972 (33.2%) | <0.001 |
| Overall health rating |  |  |  |  |  |  | <0.001 |
| Excellent | 67,413 (17.9%) | 13,307 (17.6%) | 13,233 (17.6%) | 13,691 (18.1%) | 13,520 (17.9%) | 13,662 (18.1%) |  |
| Good | 224,017 (59.4%) | 44,621 (59.0%) | 44,637 (59.3%) | 44,632 (58.9%) | 45,573 (60.4%) | 44,554 (59.2%) |  |
| Fair | 73,088 (19.4%) | 15,050 (19.9%) | 14,813 (19.7%) | 14,718 (19.4%) | 13,949 (18.5%) | 14,558 (19.3%) |  |
| Poor | 12,822 (3.4%) | 2,685 (3.5%) | 2,556 (3.4%) | 2,682 (3.5%) | 2,384 (3.2%) | 2,505 (3.3%) |  |
| Family history of heart diseases | 144,592 (38.3%) | 29,656 (39.2%) | 28,273 (37.6%) | 28,835 (38.1%) | 29,158 (38.7%) | 28,670 (38.1%) | <0.001 |
| Hypertension | 89,704 (23.8%) | 18,032 (23.8%) | 18,012 (23.9%) | 18,079 (23.9%) | 17,694 (23.5%) | 17,887 (23.8%) | 0.214 |
| Diabetes | 13.626 (3.6%) | 2,331 (3.1%) | 2,802 (3.7%) | 2,983 (3.9%) | 2,438 (3.2%) | 3,072 (4.1%) | <0.001 |
| Aspirin | 36,598 (9.7%) | 7,254 (9.6%) | 7,334 (9.7%) | 7,198 (9.5%) | 7,372 (9.8%) | 7,440 (9.9%) | 0.095 |
| Anti-hypertension medicine | 20,667 (5.5%) | 4,282 (5.7%) | 4,208 (5.6%) | 3,915 (5.2%) | 4,055 (5.4%) | 4,207 (5.6%) | <0.001 |
| Lipid-lowering medicine | 21,138 (5.6%) | 4,268 (5.6%) | 4,497 (6.0%) | 4,532 (6.0%) | 3,965 (5.3%) | 3,876 (5.1%) | <0.001 |
| Nitrogen dioxide air pollution, microg/m^3^ | 26.7±7.6 | 34.0±6.8 | 30.4±5.9 | 27.1±5.1 | 23.6±4.3 | 18.4±4.2 | <0.001 |
| Nitrogen oxides air pollution, microg/m^3^ | 44.0±15.5 | 55.2±16.4 | 49.7±14.5 | 44.8±12.5 | 39.5±10.7 | 31.0±9.9 | <0.001 |
| PM10, microg/m^3^ | 16.2±1.9 | 16.3±1.8 | 16.4±2.0 | 16.5±1.9 | 15.9±1.7 | 16.2±1.8 | <0.001 |
| PM2.5, microg/m^3^ | 10.0±1.0 | 10.3±1.1 | 10.1±1.2 | 10.1±1.0 | 9.8±1.0 | 9.7±0.8 | <0.001 |
| Traffic intensity on the nearest road, vehicles/day | 1513.1±4933.5 | 1230.7±3968.3 | 1657.4±5047.5 | 1722.8±5925.0 | 1447.2±4493.6 | 1507.5±5001.2 | <0.001 |
| Inverse distance to the nearest road, 1/meters | 0.05±0.07 | 0.05±0.07 | 0.05±0.07 | 0.05±0.07 | 0.05±0.08 | 0.05±0.07 | <0.001 |
| Average daytime sound level of noise pollution, dB | 55.4±4.3 | 55.3±4.0 | 55.7±4.6 | 55.7±4.5 | 55.2±4.1 | 55.1±4.1 | <0.001 |
| Average evening sound level of noise pollution, dB | 51.7±4.3 | 51.6±4.0 | 52.0±4.6 | 51.9±4.5 | 51.5±4.1 | 51.4±4.1 | <0.001 |
| Average night-time sound level of noise pollution, dB | 46.6±4.3 | 46.5±4.0 | 46.9±4.6 | 46.8±4.5 | 46.4±4.1 | 46.3±4.1 | <0.001 |
| Food weight, g | 3263.1±374.0 | 3263.7±344 | 3258.4±444.3 | 3267.3±401.4 | 3260.6±330.6 | 3265.3±336.4 | <0.001 |
| Energy, KJ | 8826.7±1284.0 | 8835.3±1189.0 | 8824.8±1531.1 | 8824.1±1391.2 | 8824.3±1125.7 | 8825.1±1132.9 | <0.001 |
| Protein, g | 81.8±12.4 | 81.8±13.4 | 81.8±14.8 | 81.8±13.6 | 81.8±11.0 | 81.8±10.9 | <0.001 |
| Fat, g | 76.8±14.5 | 76.9±13.4 | 76.8±17.3 | 76.8±16.0 | 76.7±12.5 | 76.7±12.8 | <0.001 |
| Carbohydrate, g | 258.1±43.1 | 258.5±39.7 | 257.9±51.6 | 257.7±46.1 | 258.2±37.9 | 258.4±38.4 | <0.001 |
| Englyst dietary fiber, g | 16.6±3.1 | 16.7±2.9 | 16.6±3.7 | 16.6±3.4 | 16.6±2.8 | 16.6±2.7 | <0.001 |
| Sleep duration, hours/day | 7.14±1.1 | 7.15±1.1 | 7.13±1.1 | 7.13±1.1 | 7.17±1.1 | 7.14±1.1 | <0.001 |
