# Supplementary figures and images for: Association of Residential Proximity to the Coast With Incident Myocardial Infarction: A Prospective Cohort Study
Source: Front Cardiovasc Med. 2022 Feb 17;9:752964. doi: 10.3389/fcvm.2022.752964 (PMC8891518; doi:10.3389/fcvm.2022.752964)

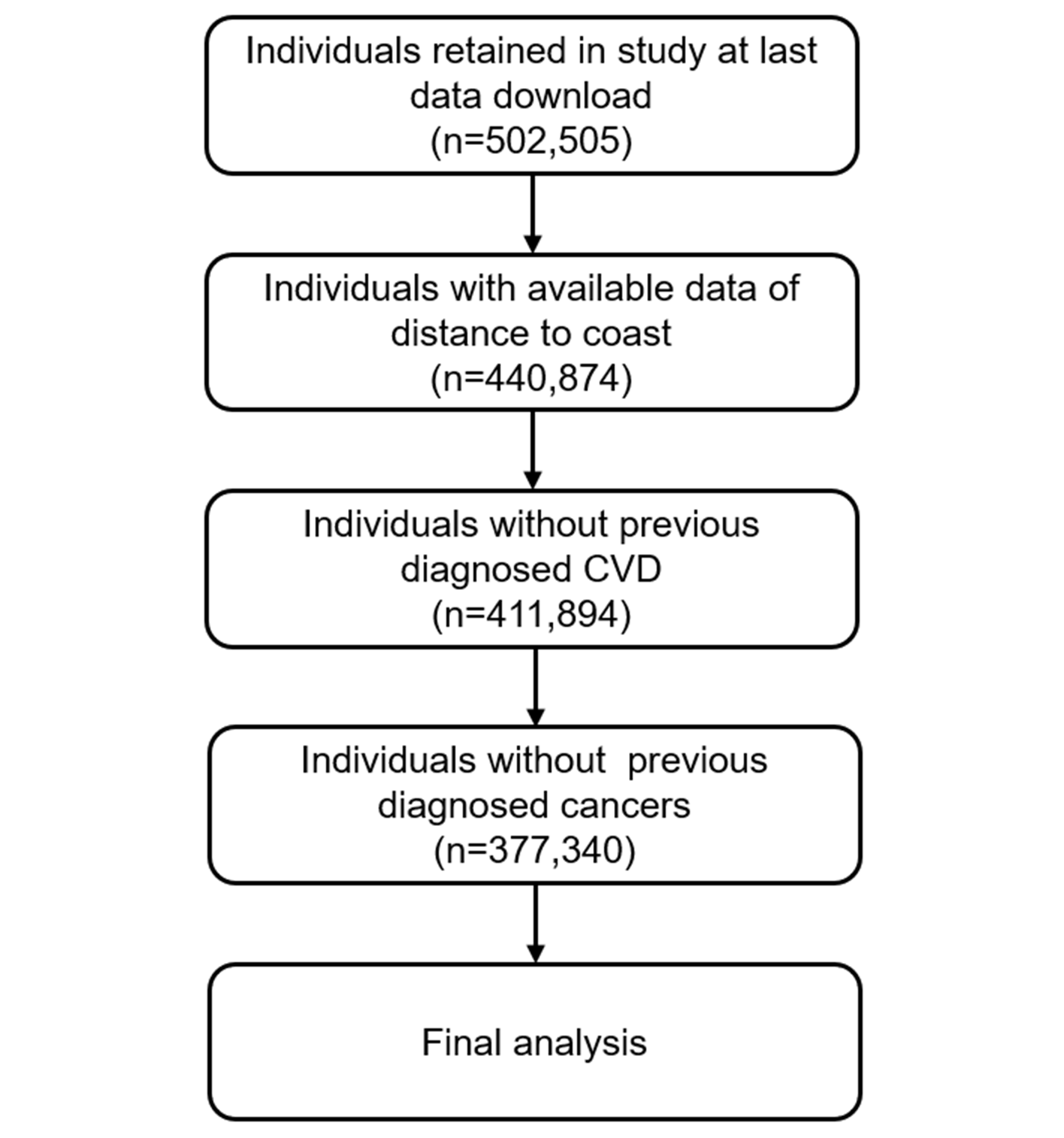

Supplement: Supplementary file 2 [file Image_1.TIF]
